# MicroRNAs 363 and 149 are differentially expressed in the maternal circulation preceding a diagnosis of preeclampsia

**DOI:** 10.1038/s41598-020-73783-w

**Published:** 2020-10-22

**Authors:** Carole-Anne Whigham, Teresa M. MacDonald, Susan P. Walker, Richard Hiscock, Natalie J. Hannan, Natasha Pritchard, Ping Cannon, Tuong Vi Nguyen, Manisha Miranda, Stephen Tong, Tu’uhevaha J. Kaitu’u-Lino

**Affiliations:** 1 Translational Obstetrics Group, The Department of Obstetrics and Gynaecology, Mercy Hospital for Women, University of Melbourne, 163 Studley Road, Heidelberg, VIC 3084 Australia; 2 Mercy Perinatal, Mercy Hospital for Women, Heidelberg, VIC Australia

**Keywords:** Biomarkers, Predictive markers

## Abstract

Preeclampsia is a pregnancy complication associated with angiogenic dysbalance, maternal endothelial dysfunction and end-organ injury. A predictive test to identify those who will develop preeclampsia could substantially decrease morbidity and mortality. MicroRNAs (miRs) are small RNA molecules involved in post-transcriptional gene regulation. We screened for circulating miRs differentially expressed at 36 weeks’ gestation in pregnancies before the development of preeclampsia. We used a case–control group (198 controls, 34 pre-preeclampsia diagnosis) selected from a prospective cohort (n = 2015) and performed a PCR-based microarray to measure the expression of 41 miRs. We found six circulating miRs (miRs 363, 149, 18a, 1283, 16, 424) at 36 weeks' had significantly reduced expression (p < 0.0001–0.04). miR363 was significantly downregulated at 28 weeks’ gestation, 10–12 weeks before the onset of clinical disease. In the circulation of another cohort of 34 participants with established preterm preeclampsia (vs 23 controls), we found miRs363, 18a, 149 and 16 were significantly down regulated (p < 0.0001–0.04). Combined expression of miRs149 and 363 in the circulation at 36 weeks’ gestation provides a test with 45% sensitivity (at a specificity of 90%) which suggests measuring both miRs may have promise as part of a multi-marker test to predict preeclampsia.

## Introduction

Preeclampsia is a hypertensive disorder of pregnancy associated with poor placental implantation and remodelling of the maternal spiral arteries. There is angiogenic dysbalance with increased antiangiogenic factors released from the placenta. These circulate widely in the maternal circulation, inciting maternal endothelial dysfunction^1,2^. Preeclampsia is a major cause of maternal and fetal morbidity and mortality, affecting 3–8% of pregnancies, and is directly associated with 15% of maternal deaths around the world^3^.

While recent studies have identified screening tests that have good sensitivity in identifying women at risk of developing preterm preeclampsia^4^, there are currently no diagnostic tests that predict those who will develop term preeclampsia. Late onset disease represents the most common form and women who are affected can rapidly develop preeclampsia with severe features. Earlier detection of women at risk could have significant impact on maternal–fetal wellbeing^5^.

MicroRNAs (miRs) are short, non-coding RNA molecules which have important functions in the post transcriptional regulation of gene expression^6^. It is thought that miRs regulate 60% of protein coding genes in the human body^7^, which highlights their central roles in driving cellular function. MiRs can be packaged inside vesicles or exosomes^8^ where they are protected from degradation by enzymes. Therefore, they are incredibly stable, making them ideal candidates for biomarker discovery^9^.

In this study we hypothesised that circulating miRs might be predictive biomarkers of late-onset preeclampsia, measured at 28 or 36 weeks’ of pregnancy. Furthermore, we suggest that there might be a common, overlapping abnormal expression of these miRs in both late onset preeclampsia and early onset preeclampsia, and investigate this further by measuring the expression of miRs in a preterm cohort.

In choosing which miRs to include in our screening panel, we first selected those from the primate specific C19MC cluster as they are primarily expressed in the placenta^10^. Given preeclampsia is principally a disease of placental dysfunction it is plausible that the expression profile of these miRs in the maternal circulation may be different in those who will go onto develop preeclampsia. We chose to investigate 32 miRs from the C19MC cluster, based on their detectability in maternal circulation and/or placental tissue^11^.

Besides the C19MC cluster, we identified three additional miRs reported to be altered in the circulation of patients with established preeclampsia^12–15^.

We measured circulating levels of a further five miRs reported as altered in preeclamptic placentas^16–18^, and finally miR144 based on evidence it is altered very early in the maternal circulation, in first trimester, before the onset of preeclampsia^19^ (see Supplementary Table S1).

These miRs were measured in a case control set of blood samples obtained at 36 weeks’ gestation using PCR based microarray technology. The miRs that were found to be differentially expressed at 36 weeks’ were then measured in samples obtained at 28 weeks’ gestation (10–12 weeks before the onset of clinical disease), and in both the blood and placentas in key cohorts of women with an established diagnosis of preeclampsia.

## Results

### Expression of miRs 18a, 363, 1283, 149, 16 and 424 at 36 weeks’ gestation are reduced among women who will go on to develop preeclampsia at 36 weeks’ gestation

To determine whether any of the 41 miRs we selected were differentially expressed in the maternal circulation at 36 weeks’ gestation among those who will develop preeclampsia, their expression was measured in samples obtained from the FLAG study.

**Figure 1:**
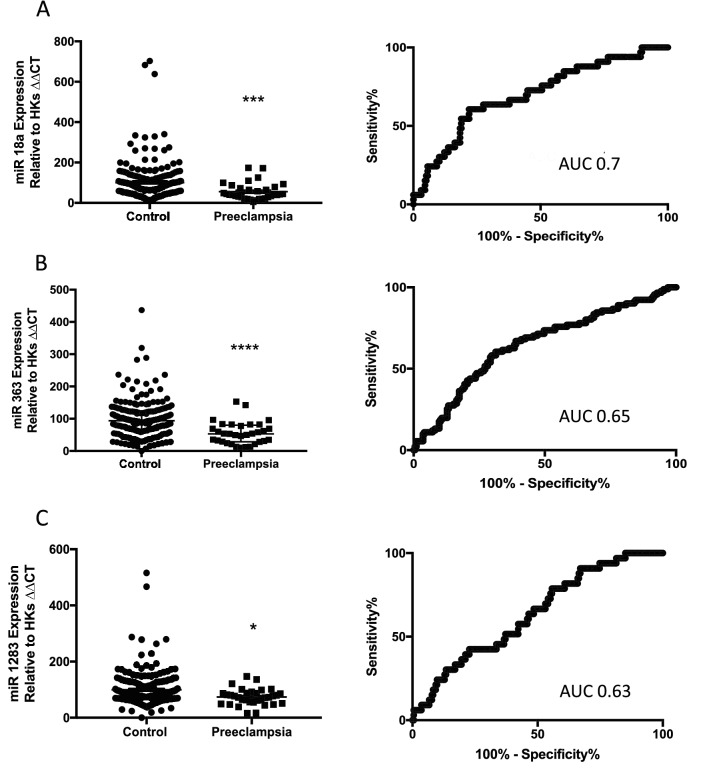

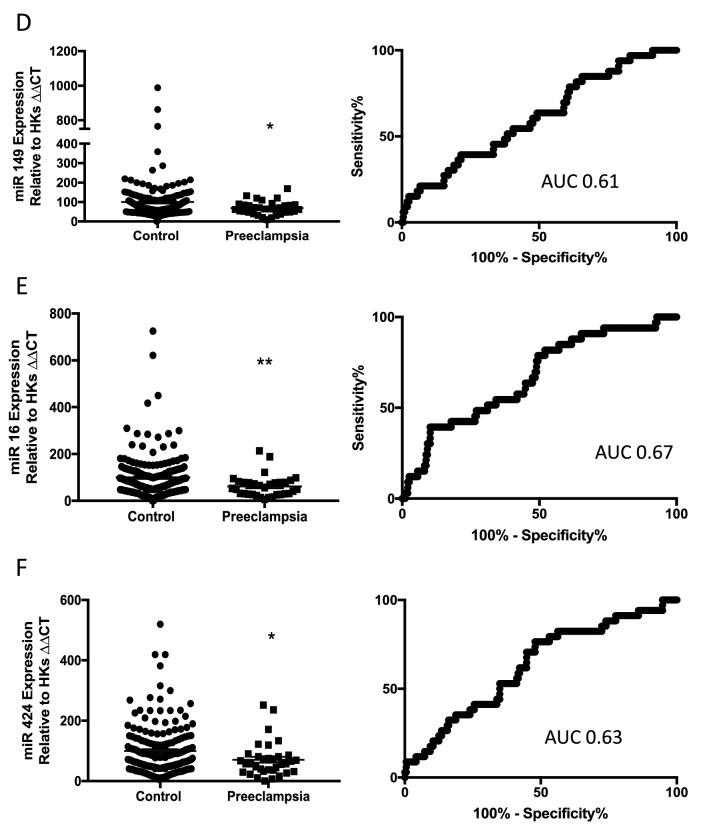
Circulating miRs are reduced in maternal whole blood in women at 36 weeks’ gestation who will go on to develop term preeclampsia. *miR18a* (**A**), *miR363* (**B**), *miR1283* (**C**), *miR149* (**D**), *miR16* (**E**) and *miR424* (**F**) were all significantly reduced in the whole blood of women who will go on to develop preeclampsia relative to control women. Area under the receiver operator curve (AUC ROC) are shown on each graph. *p < 0.05, **p < 0.01, ***p < 0.001, ***p < 0.0001. Data expressed as mean ± SEM, individual symbols represent individual patients. PE n = 34, controls n = 196.

We identified six miRs that were differentially expressed at 36 weeks’ gestation among those who will go onto develop preeclampsia compared to controls: miR18a, miR363, miR1283, miR149, miR16 and miR424 (Fig. 1A–F).

**Figure 2:**
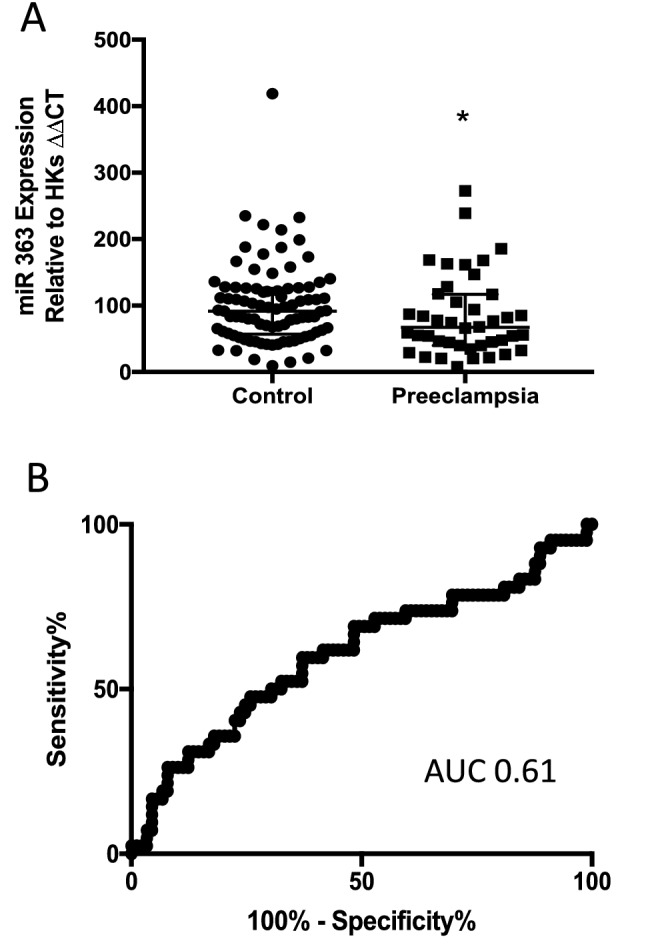
Expression of miR 363 expression at 28 weeks’ gestation in women who will go on to develop term preeclampsia. miR363 was significantly reduced in the maternal whole blood of women who will go on to develop term preeclampsia (**A**) AUC 0.61 (**B**). PE n = 43, control n = 91. *p < 0.05, **p < 0.01, ***p < 0.001, ***p < 0.0001. Data expressed as mean ± SEM, individual symbols represent individual patients.

We next measured the expression of the same six miRs in patients at 28 weeks’ gestation: 43 participants who will develop preeclampsia and 91 controls. Of the six measured, only circulating levels of miR363 were significantly reduced at 28 weeks’ gestation among participants who later develop preeclampsia (Fig. 2A), with an area under the ROC curve of 0.61 (Fig. 2B).

### Expression of miRs 18a, 363, 149 and 16 are reduced among women with established severe early onset preeclampsia

**Figure 3:**
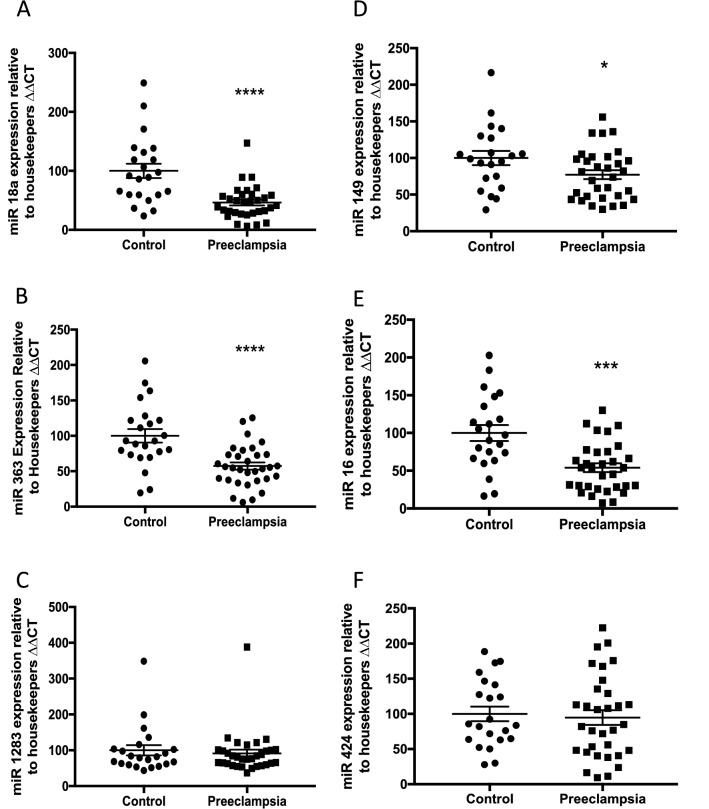
Circulating miRs in the maternal whole blood of patients with an established diagnosis of preeclampsia. *miR18a* (**A**), and *miR363* expression (**B**) are significantly reduced in the blood of patients with an established diagnosis of preeclampsia compared with gestation matched controls. *miR 1283* (**C**) expression is unchanged. *miR149* (**D**), and *miR16* (**E**) expression are significantly reduced in women with preeclampsia whilst *miR424* (**F**) expression is unchanged. *p < 0.05, **p < 0.01, ***p < 0.001, ***p < 0.0001. Data expressed as mean ± SEM, individual symbols represent individual patients. PE n = 22, controls n = 32.

We also compared the expression of the same six miRs in the circulation from a cohort of 32 women diagnosed with severe early onset preeclampsia and 22 gestation matched controls. We confirmed that four miRS were significantly decreased in the maternal circulation; miR18a, miR363, miR149 and miR16 (Fig. 3A, B, D, E). The others were not differentially expressed (Fig. 3C,F).

### Expression of miRs 363 and 149 are significantly reduced in preeclamptic placentas

**Figure 4:**
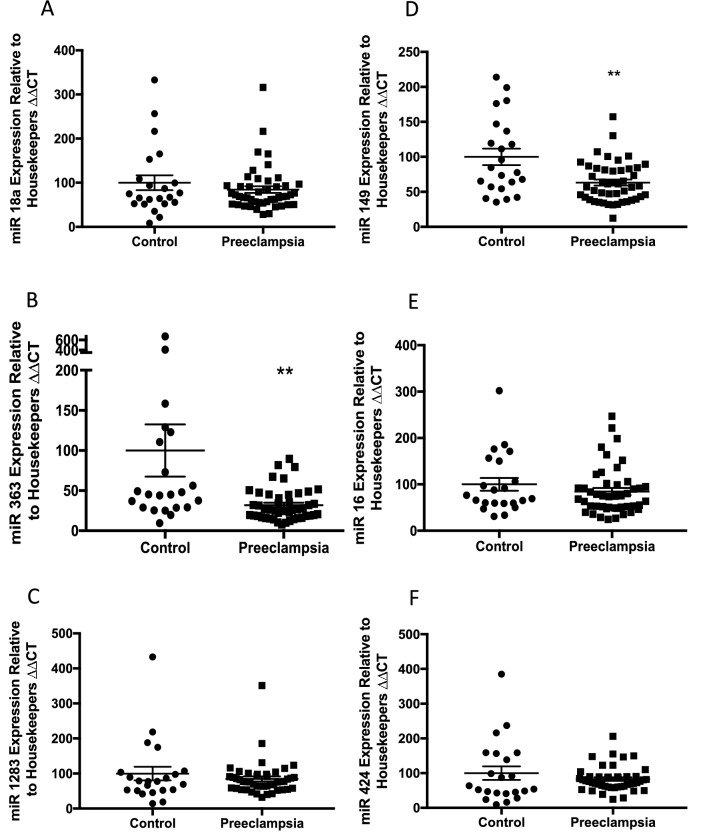
Expression of miRs in the placentas of women with an established diagnosis of preeclampsia. *miR18a* (**A**) expression was unchanged in placental tissue from women with established preeclampsia compared with gestation matched controls. Expression of *miR363* (**B**) was significantly reduced in the placentas from the preeclampsia cohort. Expression of *miR1283* (**C**) expression is unchanged. *miR149* (**D**) expression is significantly reduced in the preeclampsia cohort, whilst miR16 (**E**) and *miR424* (**F**) expression are unchanged. *p < 0.05, **p < 0.01, ***p < 0.001, ***p < 0.0001. Data expressed as mean ± SEM, individual symbols represent individual patients. PE n = 12, controls n = 34.

In order to investigate whether the altered expression observed in the maternal circulation of these six miRs originates from altered placental expression, we measured their expression in 34 preeclamptic placentas and 12 gestation matched controls. Of these, miRs 363 and 149 were significantly decreased within preeclamptic placental tissue (Fig. 4B,D). The others were not differentially expressed (Fig. 4A, C, E, F).

### Measurement of miRs in placental explants exposed to hypoxia

Placental hypoxia may be part of the pathology of preeclampsia^20^. Therefore, we exposed placental explants to hypoxia and assessed whether this altered the expression of any of the six miRs that we observed to be associated with preeclampsia. None of the six miRs examined displayed altered expression with placental hypoxia (Supplementary Fig. S1A–F)

Similarly, we isolated primary trophoblasts and exposed them to hypoxia. We measured the expression of the six miRs that were associated with preeclampsia. Of the six, only miR16 was significantly altered (Supplementary Fig. S2A–E). In contrast to the circulation at 36 weeks’ gestation expression of miR 16 was in fact increased in primary trophoblasts that were rendered hypoxic (Supplementary Fig. S2E).

### Circulating miR149 has a weak association with maternal blood pressure. miR363 levels do not correlate with maternal blood pressure at 36 weeks’ gestation, or BMI

We next sought to ascertain whether there were any significant associations between miR363 and miR149 expression, and maternal characteristics that were differentially regulated between groups, including mean arterial blood pressure (MABP) and BMI. Linear regressions were undertaken with the miR delta delta CT . As shown in supplementary Fig. S3, miR 149 had a weak association with MABP. There were no significant associations between miR149 and BMI. Further there was no association between miR363 and MABP or BMI”.

### Combining miRs 149 and 363 in the prediction of Preeclampsia

**Figure 5:**
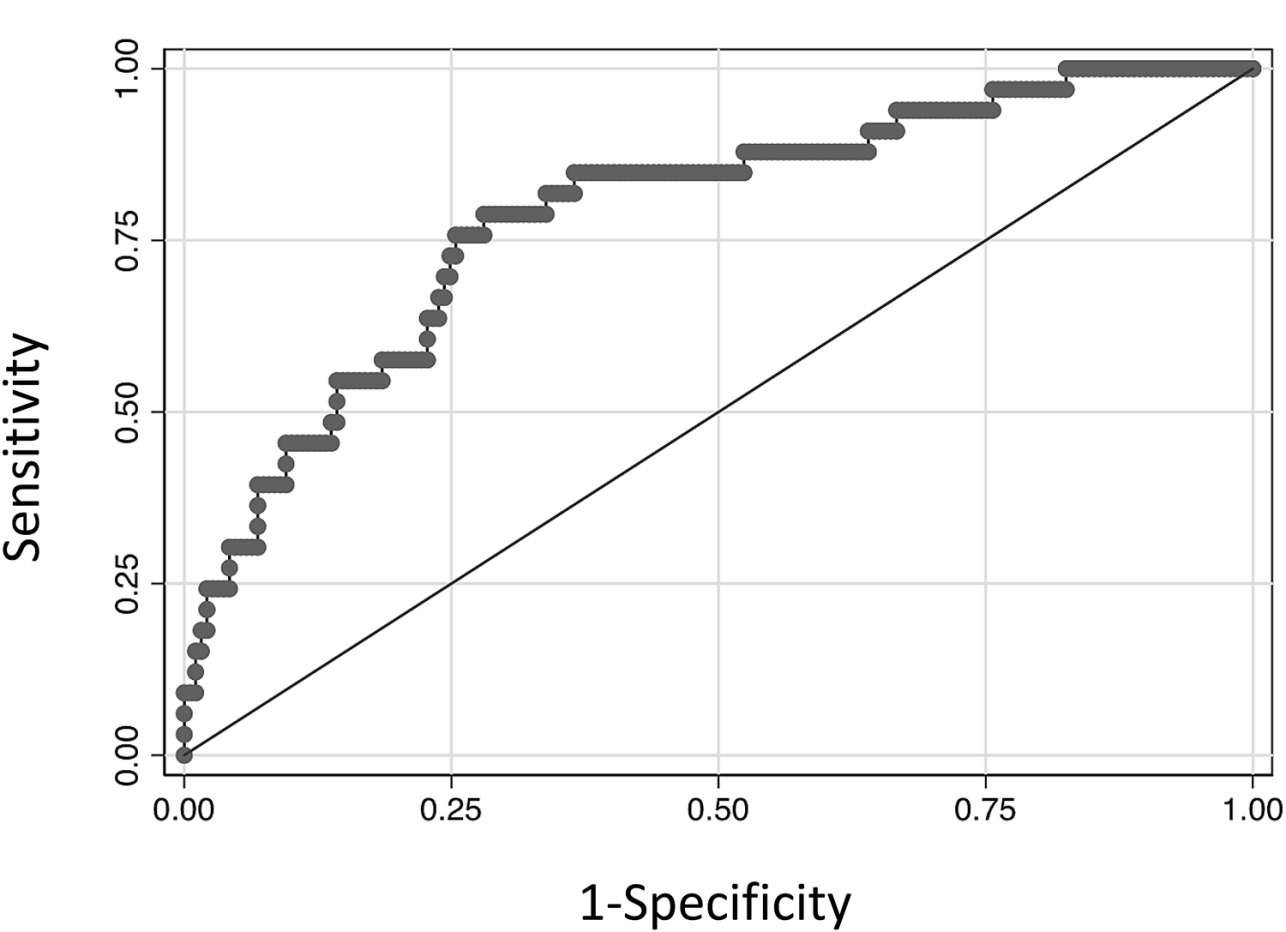
Combining miRs 149 and 363 is associated with a future diagnosis of clinical preeclampsia. At 36 weeks’ gestation, *miR149* and *miR363* combined give a predictive test with a sensitivity of 45%, specificity 90% and AUC 0.79.

Using logistic regression analysis, we found that the combination of miRs 149 and 363 derived a candidate screening test with a sensitivity of 45% (at specificity of 90%), and an area under the ROC curve of 0.79 (Fig. 5) at a cut-off of 0.2985. At a prevalence of 5%, this gives a positive predictive value of 19% and negative predictive value of 97%.

## Discussion

Developing a test to predict which patients will develop preeclampsia in pregnancy has been a hot topic in reproductive biology. Combining maternal risk factors, first trimester uterine artery pulsatility index and maternal mean arterial pressure, alongside multiple of the median (MoM) values for maternal serum pregnancy associated plasma protein-A (PAPP-A) and placental growth factor (PlGF), yielded a sensitivity of 76% in identifying those who would develop preterm preeclampsia^4,21^. However, this algorithm was far less successful at predicting those who will develop the disease at term, with a sensitivity of 43%^21^.

The poor sensitivity for tests performed in the first trimester detecting^4,21^ preeclampsia at term leaves a gap in prediction models. Term preeclampsia affects around 3% of pregnancies whereas the preterm condition affects 0.4%. Many diagnosed with term disease can also develop preeclampsia with severe features, that is significant maternal organ injury. As such, term disease can also be associated with significant morbidity^22^. Moreover, there has been a plethora of evidence demonstrating that inducing labour at term conveys no significant adverse outcomes to the mother or fetus^5,23–25^. Thus, if a test were able to identify the women at risk of developing term (or post-partum) preeclampsia, clinicians could increase antenatal and postnatal surveillance and provide tailored care to avoid the development of maternal preeclampsia, and potential severe adverse outcomes to both mother and baby.

In this study, we screened 41 miRs in the circulation of pregnant women at 36 weeks’ gestation to identify miRs that are dysregulated prior to the onset of preeclampsia. We identified six miRs that are down regulated among those who will develop term preeclampsia compared to controls. Of particular interest, circulating miR363 was consistently downregulated preceding disease diagnosis (as early as 10–12 weeks before), in women with established disease and in the placentas of women with severe early onset preeclampsia. The combination of miRs 149 and 363 derived a candidate screening test with a sensitivity of 45% (at specificity of 90%), and an area under the ROC curve of 0.79 (Fig. 5).

It is now well established that preeclampsia is a disease of severe endothelial dysfunction^26^. miR363 has been previously shown to regulate some endothelial cell properties via its post transcriptional regulation of tissue inhibitor of metalloproteinases-1^18^ (TIMP-1) and thrombospondin 3^27^. Thus, our identification of this ‘endothelial miR’ being dysregulated in the circulation of women preceding their diagnosis of preeclampsia and also within preeclamptic placentas suggests that it may regulate mRNAs important to both endothelial function and placental function. Identification of its target mRNAs within both these areas may indeed provide further insight into the disease pathogenesis.

Three other miRs were also selected for the microarray on the basis that previous studies have demonstrated that they are important in the regulation of endothelial cell function—miRs 149, 424 and 18a. MiR18a has demonstrated anti-angiogenic properties on endothelial cells and thus its downregulation may also contribute to the endothelial cell dysfunction apparent in preeclampsia^28^. miR149 has also been shown to regulate genes associated with endothelial dysfunction^29^ and its expression decreases from pre- to post-pregnancy, indicating it may be important during gestation or be produced from gestational tissues. As term preeclampsia is thought to be a disease of dysfunctional maternal endothelium and less so of poor initial placentation, it is perhaps unsurprising that we found a significant difference in these ‘endothelial dysfunction’ miRs in those who would go on to develop term preeclampsia.

In our studies we also identified circulating miR16 as significantly down-regulated in those who would go on to develop preeclampsia, and in those with established disease. Interestingly, miR16 targets vascular endothelial growth factor-A (VEGFA), a pro-angiogenic molecule that regulates placental angiogenesis and is important in normal vascular function^30^. miR 16 has previously been shown to be increased in the placentas of patients with preeclampsia^31^ and down regulated in those with a baby that is small for gestational age^32^. Thus, we hypothesise that its down regulation in the circulation may relate to the anti-angiogenic state of preeclampsia, for which further impairment of pro-angiogenic molecules is not needed, or indeed may be detrimental.

We were surprised to find that only one of the miRs that were selected from the C19MC cluster was significantly altered in those who would go on to develop preeclampsia, given their distinct expression within placenta^33^. miR1283 has been shown to be vital for trophoblast proliferation during the first trimester^34^. However, miR1283 also regulates activating transcription factor 4 (ATF4) which is important in maintaining function in primary human umbilical vein endothelial cells. So, although the C19MC cluster are primarily expressed in the placenta, it is clear that members of the cluster are expressed in other tissues. Thus, potentially it is the endothelial cell dysfunction characteristic of term preeclampsia, rather than placental release, that is responsible for the change in miR1283 expression in patients who would go on to develop preeclampsia.

Our second hypothesis is that there might be common, overlapping abnormal expression of these miRs in both late onset preeclampsia and early onset preeclampsia. In our cohort of patients with an established diagnosis of early onset preeclampsia we found that four of the miRs were differentially expressed. This indicates that the pathology leading to the imbalance of these miRs is occurring in both early and late onset disease. In late onset disease, it has been suggested that the main pathophysiological process is endothelial dysfunction with a lesser emphasis on abnormal placentation as it seen in the early onset form of the disease^35^. However in early onset disease there exists significant endothelial dysfunction^36^. This may suggest that endothelial dysfunction is a contributing factor to the abnormal expression of these miRs. However, we note that we were unable to find any correlation between circulating miR363 expression and MABP at 36 weeks’ gestation. We found an inverse correlation between with miR149 and MABP at 36 weeks gestation. The very modest positive association between miR149 and MABP is not in keeping with this miR playing a significant role in BP changes in preeclampsia.

We have screened a large panel of circulating miRs at 36 weeks’ gestation in those who will go on to develop preeclampsia. Interestingly, the miRs found to be most consistently dysregulated were those that are implicated in endothelial cell dysfunction, in particular miR363 which was down-regulated as early as 28 weeks’ gestation. Importantly, we show that miRs363 and 149 at 36 weeks’ gestation are significantly associated with a future diagnosis of clinical preeclampsia and provide a test with a better sensitivity and specificity than others for term disease. In the future, we hope to combine these miRs with other analytes, including possibly sFlt-1 and PlGF, to formulate a sensitive and specific test to predict those patients who are predicted to develop term preeclampsia. In addition, identification and understanding of the mRNAs that are regulated by these miRs may provide further insight into the pathogenesis of this disease.

## Methods:

### Blood and tissue collection ethical approval

This study was approved by the Mercy Health Research Ethics Committee (Ethics Approval Number R14/12, R11/34) and written informed consent was obtained from all participants. All methods were performed in accordance with the relevant guidelines and regulations^37^.

### *FLAG study design overview*^38^

This study is part of the Fetal Longitudinal Assessment of Growth (FLAG) study Women carried out at the Mercy Hospital for Women, a tertiary maternity hospital in Melbourne with approximately 6000 births annually. The FLAG study, designed to identify biomarkers to detect small for gestational age (SGA) fetuses, included prospective collection of 2015 blood samples from pregnant women at 28 and 36 weeks’ gestation (collected Feb 2015-May 2016). 3.9% of patients developed preeclampsia^37^.

We performed a case–control study using blood samples chosen from the first 1000 FLAG participants. We compared the 36 and 28 week miR values from women who subsequently developed preeclampsia after 36 weeks’ gestation, to the analyte levels in gestation matched blood samples from a cohort of randomly selected controls. We chose controls from those with a well grown fetus (i.e. birthweight > 10th%) where the mother had no preeclampsia at any point during the antenatal or postnatal period. At 36 weeks’ gestation, we analysed 198 control samples (Demographics Supplementary Table S2) and 34 samples from women who will go on to develop preeclampsia. Six women had a pregnancy complicated by fetal growth restriction as well as preeclampsia. Of the samples collected at 28 weeks (Demographics Supplementary table S3), 91 controls were compared to 43 samples from those who will go on to develop preeclampsia^37^.

### FLAG recruitment

Women were screened for eligibility and invited to participate at their oral glucose tolerance test, offered to all women around 28 weeks’ gestation to diagnose gestational diabetes (part of routine care). English-speaking women aged over 18 years, carrying a well-dated singleton pregnancy with normal mid-trimester morphology ultrasound were eligible to participate. Whole blood was collected in a PAXgene RNA tube at the time of enrolment, i.e. 27^+0^–29^+0^ weeks and/or at 35^+0^ to 37^+0^ weeks’ gestation inclusive^38^.

### Outcomes and diagnostic criteria

Maternal characteristics and pregnancy outcomes were obtained by review of each participant’s medical record, investigation results and hospital database entry (Supplementary table S2 for 36 week PE characteristics and demographics, and Supplementary Table S3 for 28-weeks’ gestation sample demographics).

Preeclampsia was diagnosed as defined by The American College of Obstetricians and Gynecologists’ Taskforce on Hypertension in Pregnancy^39^, new onset hypertension (blood pressure ≥ 140 mmHg systolic, or ≥ 90 mmHg diastolic on two occasions ≥ 4 h apart after 20 weeks’ gestation); plus one or more of the following: proteinuria, thrombocytopaenia, renal insufficiency, impaired liver function, pulmonary oedema or cerebral symptoms^37^.

### Established preeclampsia—cases and controls: blood collection

We obtained 22 blood samples from women with preterm pregnancies not complicated by preeclampsia, and 32 samples from women with severe early-onset preeclampsia. Preeclamptics were diagnosed in accordance with the American College of Obstetrician and Gynecologists (ACOG) guidelines 2013^39^. All samples were obtained from cases of early-onset preeclampsia (< 34 weeks’ gestation). Preterm controls were selected from women with pre-term rupture of membranes, placenta praevia or antepartum haemorrhage without any evidence of infection (histopathological examination of the placentas), hypertensive disease or maternal co-morbidities. Whole blood was collected in a PAXgene RNA tube at the time of consent. All methods were performed in accordance with the relevant guidelines and regulations^37^. All patients delivered by caesarean section. Patient characteristics are shown in Supplementary Table S4.

### Established preeclampsia—cases and controls: placental tissue collection

We obtained preterm placentas from 12 pregnancies not complicated by preeclampsia and 34 placentas from those with established severe early-onset disease to examine miR expression.

### miRNA extraction from PAXgene tubes

PAXgene blood RNA tubes were incubated for at least 2 h at room temperature after blood collection, before storage at − 80 °C according to the manufacturers instructions. For extraction, tubes were thawed and then centrifuged for 10 min at 3000–5000*g.* Supernatant was discarded and the pellet resuspended in RNase free water before another centrifuge for 10 min at 3000–5000*g*. The QIAcube (Qiagen, Valencia, CA) shaker was used as per manufacturer’s instructions to extract RNA. RNA was subsequently snap frozen and stored at – 80 °C^37^.

### Extraction of miRNA from placental tissue

The Qiagen miRNeasy mini kit, combined with the MinElute kit, were used to extract miRNA from placental tissue as per manufacturers guidelines.

### cDNA and RT-PCR

miRNA was converted to cDNA using the miScript II RT kit (Qiagen), as per manufacturer guidelines.

For the microarray, the miScript SYBR green PCR kit (Qiagen) was used. Housekeeping reference miR191, SNORD44 and SNORD48 were included in the qRT-PCR microarray, as well as 4 quality controls. Supplementary Table S1 lists the microRNAs included in the microarray. Thirty-six microRNAs were selected from the C19MC cluster and nine others from a literature search as detailed in the introduction. The microarray was designed by Qiagen and custom made for this project. Primers for the PCR were also provided by Qiagen and were designed for detected mature miRs. Housekeeper values were analysed and found to be invariable between groups.

The following conditions were used to carry out the PCR reactions:

Activation step: 95 °C for 15 min, followed by 40 cycles of: 94 °C for 15 s, 55 °C for 30 s, 70 °C for 30 s. Data were analysed using the ΔΔCT method of analysis.

### Isolation of primary human cytotrophoblast

Human cytotrophoblasts were isolated from term, caesarean section placentas as previously described^40,41^. Primary cytotrophoblasts were cultured in DMEM high Glutamax (Life Technologies) containing 10% FCS and 1% antibiotic–antimycotic on fibronectin (10 ug/mL; BD Biosciences, New South Wales, Victoria) coated wells. Cells were plated and allowed to attach over 12–18 h before washing with dPBS (Life Technologies) to remove cellular debris. Primary cytotrophoblasts were maintained in a humidified incubator at 8% O_2_ (to simulate placental normoxia) or 1% O_2_ (to simulate placental hypoxia) and 5% CO_2_ for 24 h^37^.

### Isolation of placental explants

Placental tissue explants were collected from healthy term placentas. Maternal and fetal surfaces were removed before small (1 mm^3^) explants of villous tissue were dissected. 3 villous explants were utilized per well in triplicate wells. Placental explants were maintained in a humidified incubator at 8% O_2_ (normoxia) or 1% O_2_ (hypoxia) and 5% CO_2_ for 48 h then collected for miR analysis^42^.

### Statistical analysis

Triplicate technical replicates were performed for in vitro experiments, with a minimum of three independent biological replicates performed for each in vitro study. Data was tested for normal distribution and statistically analysed as appropriate using t-tests if parametric or Mann–Whitney test if not. When three or more groups were compared, a 1-way ANOVA (for parametric data) or Kruskal–Wallis test (for non-parametric data) was used. All data is expressed as mean ± SEM or median with interquartile range for non-parametric data. P values < 0.05 were considered significant. Statistical analysis was performed using GraphPad Prism 7 software (GraphPad Software, La Jolla, CA)^37^.

The datasets generated during and/or analysed during the current study are available from the corresponding author on reasonable request.

## Supplementary information

Supplementary information

Supplementary information 2

Supplementary information  3
