# Supplementary material for: MicroRNAs 363 and 149 are differentially expressed in the maternal circulation preceding a diagnosis of preeclampsia
Source: Sci Rep. 2020 Oct 22;10:18077. doi: 10.1038/s41598-020-73783-w (PMC7583242; doi:10.1038/s41598-020-73783-w)
Supplement: Supplementary file 1 — Supplementary information [file 41598_2020_73783_MOESM1_ESM.pptx]

## Slide 1
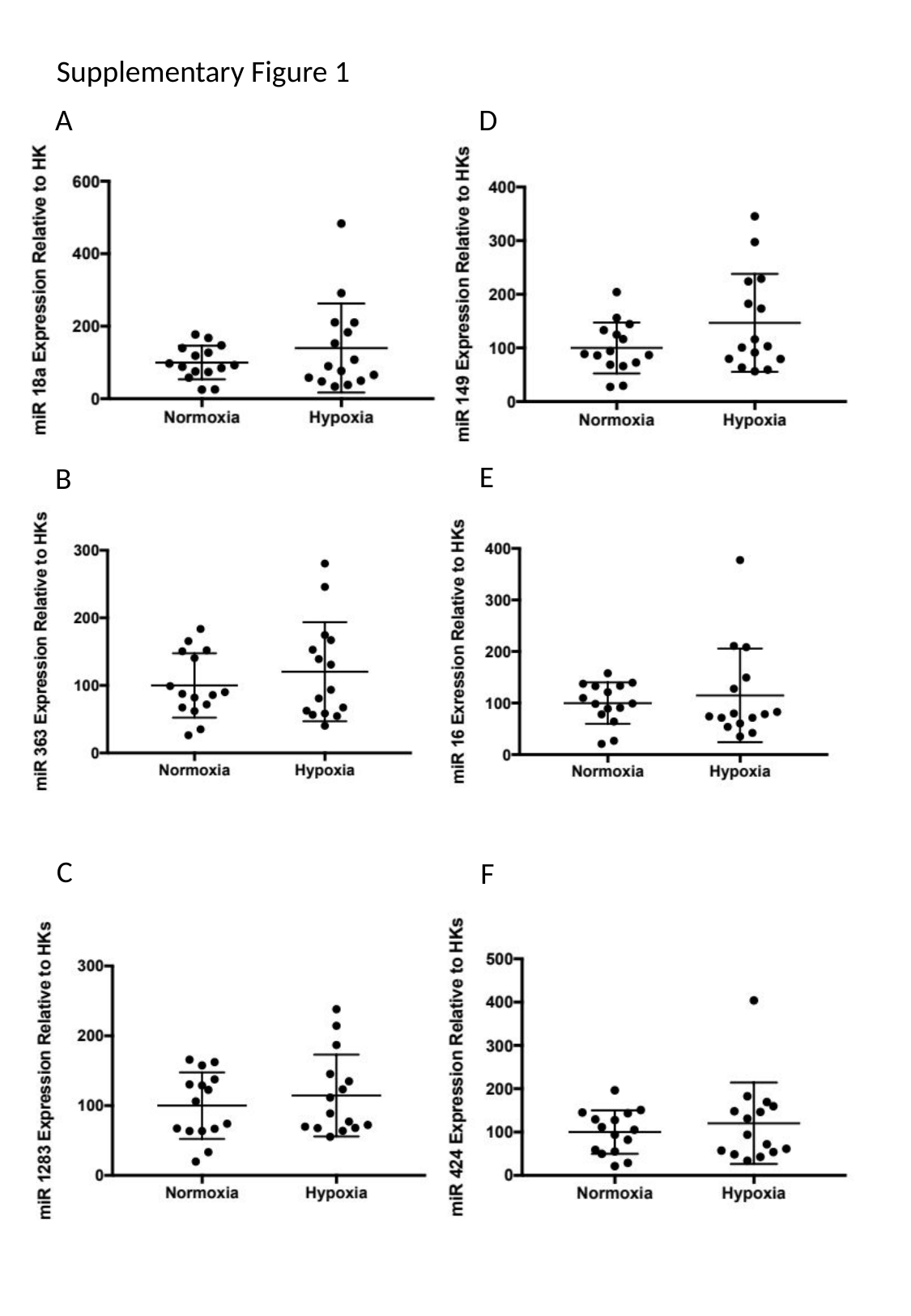

Supplementary Figure 1
A
D
E
B
C
F

## Slide 2
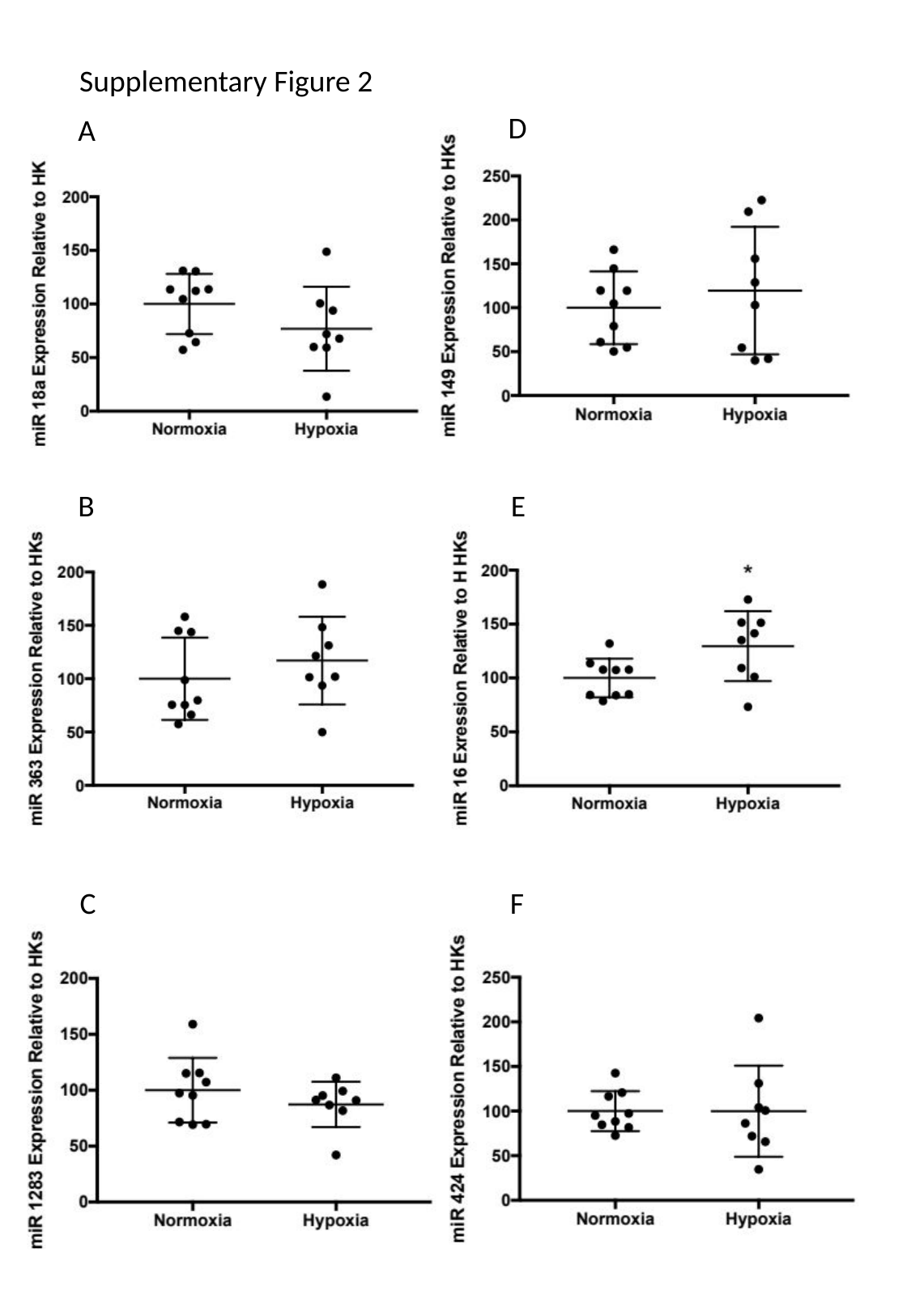

Supplementary Figure 2
D
A
B
E
C
F

## Slide 3
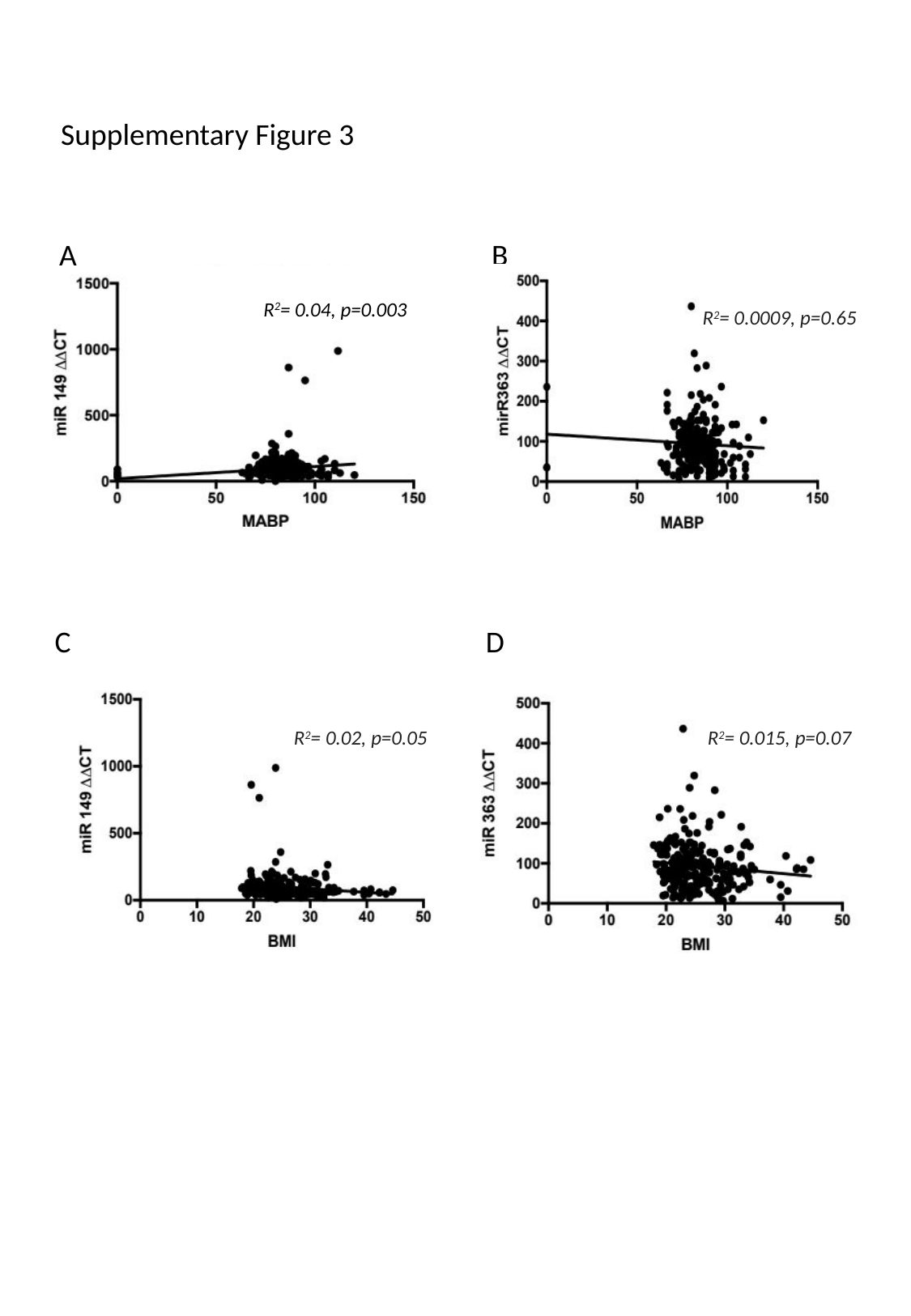

Supplementary Figure 3
A
B
R2= 0.04, p=0.003
R2= 0.0009, p=0.65
C
D
R2= 0.02, p=0.05
R2= 0.015, p=0.07
