# Supplementary material for: MicroRNAs 363 and 149 are differentially expressed in the maternal circulation preceding a diagnosis of preeclampsia
Source: Sci Rep. 2020 Oct 22;10:18077. doi: 10.1038/s41598-020-73783-w (PMC7583242; doi:10.1038/s41598-020-73783-w)
Supplement: Supplementary file 2 — Supplementary information 2 [file 41598_2020_73783_MOESM2_ESM.docx]

**MicroRNAs 363 and 149 are differentially expressed in the maternal circulation preceding a diagnosis of preeclampsia.**

**Authors**

Carole-Anne Whigham^1,2^, Teresa M MacDonald^1,2^, Susan P Walker^1,2^, Richard Hiscock^1^, Natasha Pritchard^1,2^, Natalie J Hannan^1,2^, Ping Cannon^1,2^, Tuong Vi Nguyen^1,2^, Manisha Miranda^2^, Stephen Tong^1,2^, Tu’uhevaha J Kaitu’u-Lino^1,2^

^1^ Translational Obstetrics Group, The Department of Obstetrics and Gynaecology, Mercy hospital for Women, University of Melbourne, 163 Studley Road, Heidelberg 3084, Victoria, Australia.

^2^ Mercy Perinatal, Mercy Hospital for Women, Victoria, Australia.

Corresponding Author:

Dr Carole-Anne Whigham

Mercy Hospital for Women

Dept. of Obstetrics and Gynaecology, University of Melbourne

163 Studley Road

Heidelberg, Vic 3084, Australia

Telephone: +613 8458 4377

Email: [drcwhigham@gmail.com](mailto:drcwhigham@gmail.com)

The authors report no conflicts of interest.

*Supplementary Table 1: miRS included in the microarray*

| **1** | QC | **19** | 519E | **37** | 516-1 |
| --- | --- | --- | --- | --- | --- |
| **2** | QC | **20** | 526A-1 | **38** | 516-2 |
| **3** | QC | **21** | 526A-2 | **39** | 516B-2 |
| **4** | QC | **22** | 526B | **40** | 10B |
| **5** | HK 191 | **23** | 520A | **41** | 363 |
| **6** | HK SNORD44 | **24** | 520C | **42** | 149 |
| **7** | HK SNORD48 | **25** | 520F | **43** | 16 |
| **8** | 518B | **26** | 520G | **44** | 21 |
| **9** | 518C | **27** | 520H | **45** | 424 |
| **10** | 518E | **28** | 521-1 | **46** | 144 |
| **11** | 518F | **29** | 521-2 | **47** | 29A |
| **12** | 517A | **30** | 512-1 | **48** | 18A |
| **13** | 517B | **31** | 512-2 |  |  |
| **14** | 517C | **32** | 515-1 |  |  |
| **15** | 519A-1 | **33** | 515-2 |  |  |
| **16** | 519A-2 | **34** | 523 |  |  |
| **17** | 519B | **35** | 524 |  |  |
| **18** | 519D | **36** | 525 |  |  |

*Supplementary Table 2: FLAG 36 Week Demographics*

|  | Controls (n=198) | Preeclamptic  (n=34) |
| --- | --- | --- |
| **Maternal Age** (years)  Mean | 32.48 | 32.79 |
| **Gestation at Delivery** (weeks)  Mean | 39.52 | 39.12 |
| **Gestation at Blood Collection** (weeks)  Median (IQR) | 36.14  (35.7-36.6) | 36.14  (35.6-36.5) |
| **BMI** (kg/m^2^)  Median (IQR)* | 24  (21.6-27.6) | 26.6  (23.6-30.7) |
| **Parity** % (no)  0  1  ≥2 | 62 (122)  28 (54)  10 (20) | 71 (24)  26 (9)  3 (1) |
| **Birth weight** (g)  Mean | 3466 | 3331 |
| **Male %**. (no) | 47 (93) | 47 (16) |
| **36 week Systolic BP (mmHg)**  Mean^****^ | 112^#^ | 126^#^ |
| **36 week Diastolic BP (mmHg)**  Mean^****^ | 68^#^ | 79^#^ |

# BP data available for 197/198 controls; 32/34 PE

*Supplementary Table 3: FLAG 28 Week Demographics*

|  | Controls (n=91) | Preeclamptic  (n=43) |
| --- | --- | --- |
| **Maternal Age** (years)  Mean | 32.2 | 32.8 |
| **Gestation at Delivery** (weeks)  Mean | 39.8 | 38.9 |
| **Gestation at Blood Collection** (weeks)  Mean | 28.1 | 28.0 |
| **BMI** (kg/m^2^)  Median (IQR) | 25.5  (22.3-29.3) | 27.4  (23.1-30.1) |
| **Parity** % (no)  0  1  ≥2 | 52 (47)  34 (31)  14 (13) | 74 (32)  19 (8)  7 (3) |
| **Birth weight** (g)  Median (IQR)^****^ | 3560  (3260-3800) | 2990  (2720-3390) |
| **Male %**. (no) | 48 (44) | 47 (20) |

BMI = body mass index, SBP = systolic blood pressure and DBP = diastolic blood pressure. Mann-Whitney U tests used for comparison of medians with interquartile range. ***p<0.0001 § Normal range <0.03

| *Supplementary Table 4. Maternal clinical characteristics – established preeclampsia bloods* | | |
| --- | --- | --- |
|  | Controls (n=22) | Preeclamptic  (n=32) |
| **Maternal Age** (years)  Median (IQR) | 32 (29 – 34.8) | 31 (26 – 34) |
| **Gestation at Delivery** (weeks)  Median (IQR) *** | 39.6 (39 – 40.7) | 30.6 (28 – 32) |
| **Gestation at Blood Collection** (weeks)  Median (IQR) | 28.1 (27.4 – 30.3) | 29.1 (27.7 – 30.9) |
| **BMI** (kg/m^2^)  Median (range) * | 24.7 (22 – 29.8) | 29.7 (25 – 35.2) |
| **Parity** no. (%)  0  1  ≥2 | 11 (50)  7 (32)  4 (18) | 25 (76)  5 (15)  3 (9) |
| **SBP at Booking** (mmHg)  Median (IQR) | 108 (100 -115) | 120 (110 – 125) |
| **SBP at Delivery** (mmHg)  Median (IQR) *** | 130 (125 – 132) | 170 (160 – 180) |
| **DBP at Delivery** (mmHg)  Median (IQR) *** | 79 (71 – 84) | 100 (95 – 110) |
| **Birth weight** (g)  Median (IQR) *** | 3335 (3165 – 3668) | 1127 (859 – 1531) |
| **Male** no. (%) | 14 (64) | 22 (67) |
| **Highest protein creatinine ratio (g/mmoL) §**  Median (IQR) | ---- | 0.3 (0.15 – 0.57) |
| **Liver function abnormalities**  Number (%) | --- | 19 (58) |
| **Thrombocytopenia <150 (x10^9/L)**  Number (%) | --- | 14 (42) |

BMI = body mass index, SBP = systolic blood pressure and DBP = diastolic blood pressure. Mann-Whitney U tests used for comparison of medians. BMI data available for 31/33 PE patients. SBP at booking available at booking available for 16/33 PE patients. *p<0.05 ***p<0.0001 § Normal range <0.03

| *Supplementary Table 5. Maternal clinical characteristics – placentas* | | |
| --- | --- | --- |
|  | Controls (n=12) | Preeclamptic  (n=37) |
| **Maternal Age** (years)  Median (IQR) | 31.0 (26.5 – 36.3) | 31 (28 – 33) |
| **Gestation at Delivery** (weeks)  Median (IQR) | 29.9 (28.9 – 31.6) | 30 (27.7 – 31.6) |
| **BMI** (kg/m^2^)  Median (range) | 26.2 (23.7 – 31.3) | 27 (24.3 – 37.2) |
| **Parity** no. (%)  0  1  ≥2 | 3  5  4 | 24  8  5 |
| **SBP at Booking** (mmHg)  Median (IQR) | 110 (110 – 120) | 120 (110 – 124) |
| **SBP at Delivery** (mmHg)  Median (IQR) | 120 (112 – 126) | 175 (160 – 182) |
| **DBP at Delivery** (mmHg)  Median (IQR) *** | 71 (70 – 78.5) | 100 (95 – 110) |
| **Birth weight** (g)  Median (IQR) * | 1401 (1232 – 1651) | 1094 (848 – 1400) |
| **Male** no. (%) | 6 (50) | 17 (46) |
| **Highest protein creatinine ratio (g/mmoL) §**  Median (IQR) | N/A | 0.25 (0.13 – 0.55) |
| **Liver function abnormalities**  Number (%) | N/A | 23 (62) |
| **Thrombocytopenia <150 (x10^9/L)**  Number (%) | N/A | 14 (34) |

BMI = body mass index, SBP = systolic blood pressure and DBP = diastolic blood pressure. Mann-Whitney U tests used for comparison of medians. BMI data available for 8/12 PT controls and 38/46 PE patients. SBP at booking available for 11/22 PT controls and 30/37 PE patients. *p<0.05 ***p<0.0001 § Normal range <0.03
