# Supplementary material for: MicroRNAs 363 and 149 are differentially expressed in the maternal circulation preceding a diagnosis of preeclampsia
Source: Sci Rep. 2020 Oct 22;10:18077. doi: 10.1038/s41598-020-73783-w (PMC7583242; doi:10.1038/s41598-020-73783-w)
Supplement: Supplementary file 3 — Supplementary information 3 [file 41598_2020_73783_MOESM3_ESM.docx]

**Supplementary figures:**

**Figure 1: miRs in placental explants treated in normoxia compared with hypoxia.** *miR18a* (A), *miR363* (B) *miR1283* (C) *miR149* (D) *miR16* (E) and *miR424* (F) were not significantly changed in placental explants treated in normoxia vs hypoxia. N=5.

**Figure 2: miRs in primary trophoblast cells treated in normoxia compared with hypoxia.** *miR18a* (A), *miR363* (B), *miR1283* (C) and *miR149* (D) were unchanged in primary trophoblast cells treated in normoxia compared with hypoxia. *miR16* (E) was significantly upregulated. *miR424* (F) was unchanged. *p<0.05, **p<0.01, ***p<0.001, ***p<0.0001. n=3.

**Figure 3 Circulating miR149 has a weak association with MABP. MiR363 levels do not correlate with maternal blood pressure at 36 weeks’ gestation, or BMI**

miR149 was significantly associated with MABP (A). There was no association between miR149 and BMI (C). There were also no associations between miR363 and either MABP (B), or BMI (D).
